# Stop Thinking: An Experience Sampling Study on Suppressing Distractive Thoughts at Work

**DOI:** 10.3389/fpsyg.2020.01616

**Published:** 2020-07-28

**Authors:** Cornelia Niessen, Kyra Göbel, Jonas W. B. Lang, Ute Schmid

**Affiliations:** ^1^Department of Psychology, Work and Organizational Psychology Unit, Friedrich-Alexander University of Erlangen-Nürnberg, Erlangen, Germany; ^2^Department of Personnel Management, Work and Organizational Psychology, Faculty of Psychology and Educational Sciences, Ghent University, Ghent, Belgium; ^3^Information Systems and Applied Computer Science, University of Bamberg, Bamberg, Germany

**Keywords:** suppression-induced forgetting, thought control, self-control, time pressure, experience sampling

## Abstract

In modern work environments, it can be difficult for workers to avoid becoming distracted from their current task. This study investigates person–situation interactions to predict thought control activities (kind of self-control), which aim to stop distracting thoughts that enter the mind. Specifically, it was examined (1) how challenging work demands (time pressure, task complexity) activate workers’ thought control to stop distractive thoughts (*n*_level__2_ = 143) and relate to the effort to do so (*n*_level__2_ = 91) in daily working life and (2) how these relationships differ according workers’ general cognitive ability to suppress unwanted thoughts. To understand these person–situation interactions, an experience sampling study was combined with a laboratory task assessing the ability to suppress unwanted thoughts (think/no-think task). Multilevel modeling revealed that workers’ engage more often and more intensively in thought control activities at a moderate level of time pressure but only when they had a higher general ability to suppress unwanted thoughts. For workers with a lower ability to suppress unwanted thoughts, increasing time pressure was negatively related to thought control activities, even at very low levels of time pressure. Thus, whether time pressure activates or hinders thought control depends on individuals’ ability to suppress distractive thoughts.

## Introduction

Struggling with distracting thoughts while attempting to focus on an important task is a frustrating or even stressful everyday experience at work. More than 86% of the adults in the United States reported to be constantly distracted by electronic devices, which was associated with higher stress levels (American Psychological Association, 2017). Up to 50% of an individual’s daily thoughts are not related to the ongoing task (Killingsworth and Gilbert, 2010). Surrounded by large amounts of digitalized information, and with multiple projects and tasks, workers’ performance increasingly depends on the exertion of thought control, specifically on stopping distracting thoughts that would otherwise interfere with successful task accomplishment (Schmidt and Neubach, 2007; Dabbish et al., 2011; Randall et al., 2014).

Although stopping distracting thoughts can be very difficult at times, research on self-control (e.g., Tangney et al., 2004; Schmeichel, 2007) and on motivated forgetting (Bjork et al., 1998; Anderson and Green, 2001; Anderson and Hanslmayr, 2014) has shown that individuals are capable of suppressing such undesired impulses and behaviors and that they are even able to deliberately limit access to information in memory that is emotionally distressing, unwanted, or irrelevant to task processing. Successfully stopping these unwanted thoughts and impulses can have several immediate positive consequences such as a better mood, more concentration to the task at hand, more learning, and better performance (Kanfer and Heggestad, 1999; Nørby, 2015).

However, it has also been consistently shown that the ability to inhibit unwanted and distracting memory contents differs across individuals (e.g., Levy and Anderson, 2008; Miyake and Friedman, 2012; Tangney et al., 2004) and can be compromised by situational factors (e.g., a concurrent task, Schmeichel, 2007). To explain these variations, studies have primarily focused on the underlying executive control processes (e.g., Anderson and Green, 2001; Anderson and Huddleston, 2012; Anderson and Hanslmayr, 2014) or on capacity issues such as resource depletion (e.g., Baumeister et al., 1994; Kotabe and Hofmann, 2015; Lian et al., 2017). In contrast to research on failed control, there is surprisingly little known about the activation of self-control processes (Kotabe and Hofmann, 2015; Lian et al., 2017).

In the present study, we investigated the deliberate and conscious activation of self-control processes. We focused on the activation of one specific self-control strategy, namely, on thought control that aims at limiting access to distractive memory contents to protect purposeful, goal-directed behavior. Building on previous research, we proposed that the activation of thought control in a given situation is not only a function of individuals’ ability to suppress unwanted thoughts (dispositional aspect) but also of situational demands, which foster or hinder the engagement in (state) thought control. It is important to note that we investigated both the ability to suppress unwanted thoughts (dispositional aspect) as a predictor and moderator variable and the momentary activation of thought control as an outcome variable.

**FIGURE 1:**
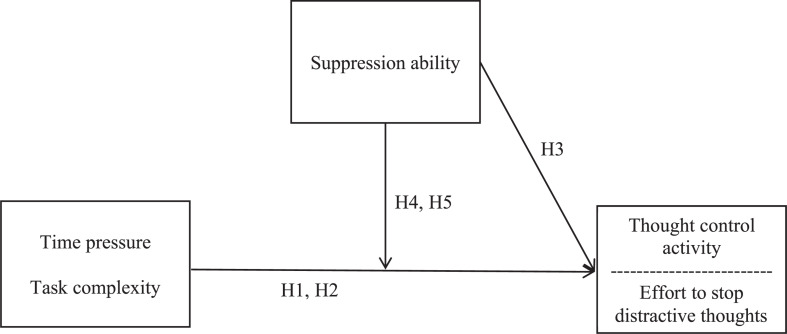
Relationships between the study’s variables.

We combined a laboratory task assessing workers’ ability to suppress unwanted thoughts, the think/no-think task (Anderson and Green, 2001), with an experience sampling study to examine how challenging work demands foster or hinder stopping distractive thoughts (i.e., state thought control) in daily working life. Specifically, we investigated two prominent challenging demands at work (Cavanaugh et al., 2000; LePine et al., 2005): time pressure (i.e., a high amount of work must be completed in little time; Spector and Jex, 1998) and task complexity (i.e., a high number of task elements and their relationships; Maynard and Hakel, 1997). Figure 1 shows the proposed relationships between the study’s variables.

The study contributes to the literature on self-control processes at work in the following ways. First, previous research on self-control has mainly concentrated on depleted self-control capacity as primary source of failed impulse and behavioral control (e.g., Baumeister et al., 1994). As this is only one aspect of the self-control process (Kotabe and Hofmann, 2015; Lian et al., 2017), we also emphasize the deliberate activation of thought control as one self-control strategy in a naturalistic setting.

Second, by combining a laboratory study with experience sampling, we were able to examine person–situation interactions, specifically, how the ability to suppress unwanted thoughts helps to keep distractive thoughts at bay in different situations in daily working life. In line with stress research, individuals’ ability to suppress unwanted thoughts can be seen as a personal resource, which can protect against the adverse effects of challenging situational demands (Hobfoll, 2002; Schmidt and Neubach, 2007). We add to this research, first, by investigating a specific ability (inhibition) rather than broader concepts such as self-control that comprises both the efforts to stimulate desirable responses and to inhibit unwanted responses (e.g., Baumeister et al., 1994; Tangney et al., 2004). Second, we measured the ability to suppress unwanted thoughts with a theoretically grounded, rigorous experimental task, the think/no-think paradigm (e.g., Anderson and Green, 2001) that allows us to overcome measurement limitations related to self-report (e.g., measure of self-control, Tangney et al., 2004; measure of thought control, Wells and Davies, 1994) often used in applied research. Finally, the intraindividual design allowed us to examine the links between challenge demands and stopping distractive thoughts on a moment-to-moment basis.

## Thought Control: Stopping Distractive Thoughts

The phenomenon of stopping distractive thoughts has been studied in different psychological subdisciplines, using different terms (Hofmann et al., 2012) such as memory control and intentional forgetting (cognitive psychology), self-control, and self-regulation (social psychology). There is considerable evidence that, in organizational settings, successful (state) self-control promotes a broad range of positive employee responses (e.g., Lian et al., 2017).

In the present study, we focus on thought control activities that aim at limiting access to distractive memory contents, thus stop distractive thoughts, to protect purposeful, goal-directed behavior.

There is considerable evidence that thought control activities such as stopping unwanted thoughts relies on inhibition (Conway et al., 2000; Mecklinger et al., 2009; Román et al., 2009; Anderson and Huddleston, 2012; Benoit et al., 2015), an essential process of executive control (see also Miyake et al., 2000; von Hippel and Gonsalkorale, 2005; Schmeichel, 2007; Miyake and Friedman, 2012). As the capacity of executive control is limited (Schmeichel, 2007; Levy and Anderson, 2008), competing tasks that require effort and attention (Ward and Mann, 2000; Lavie et al., 2004) or a previous task that require control capacity (Muraven and Baumeister, 2000; Schmeichel, 2007) can temporarily impair executive control with negative consequences for the suppression of unwanted thoughts (e.g., Schmeichel, 2007; Román et al., 2009; Ortega et al., 2012; Noreen and de Fockert, 2017). For example, Noreen and de Fockert (2017) have found that suppression of memories, assessed with the think/no-think task (Anderson and Green, 2001), suffered when participants simultaneously performed a high working memory load task. Thus, based on this research, situational demands involving heightened information processing and executive contdrol resources such as high time pressure and task complexity should make thought control activities less successful, and more difficult. Consequently, one would predict that with increasing time pressure and task complexity, workers activate thought control activities less likely, and if they do, they have to try harder.

In contrast, another line of research has revealed meta-analytic evidence that challenging situations at work (e.g., time pressure) are positively associated with performance, a result that is explained by motivational processes (LePine et al., 2004, 2005). As challenging demands are perceived as positive and changeable, individuals mobilize more effort to perform their tasks (LePine et al., 2005). This is in line with Hockey (1997) supposition that stress can increase effort and concentration on dealing with the tasks at hand. Supporting this notion, Rodell and Judge (2009) and Kane et al. (2007) showed in diary studies that persons paid more attention to challenging demands at work.

However, not all studies found a linear relationship between challenge demands and a range of employee responses; a few studies have also found an inverted U-shaped relationship (e.g., Baer and Oldham, 2006; Hofmans et al., 2015): Challenge demands increase performance but only up to a point. Researchers often draw on activation theory (Gardner, 1986; Gardner and Cummings, 1988) to explain this curvilinear relationship. Activation increases with increasing challenge demands, with positive consequences for cognitive and behavioral responses. At moderate levels of activation, individuals are optimally stimulated and increase their effort, which results in better use of cognitive resources, performance, less negative affect, and an increase in positive affect. Too little or too much activation result in less positive responses. In line with this reasoning, we assume that when working under little time pressure or accomplishing a simple task, workers may have enough cognitive capacity to think about unwanted, task-irrelevant issues while still performing well. Activation of thought control is not necessary here. Alternatively, workers might feel understimulated and thus too passive to counteract their distractive thoughts by suppressing them or even welcome distractions from a rather boring task.

As time pressure and task complexity increase, it becomes more difficult to stay focused, and therefore, workers need to increase the number of attempts to suppress irrelevant or unwanted thoughts (i.e., to engage in thought control) and the effort to stop distractive thoughts. After a certain point, however, the high working memory load resulting from high time pressure or task complexity may compete for cognitive control resources with memory suppression, leading to an overtaxing of this resource, with potential costs in terms of performance decrements. Consequently, attempts to stop distractive thoughts will decrease. Thus, after a certain point, the cost of activating thought control might outweigh its benefits for staying focused and performing well (Botvinick and Braver, 2015). Based on these theoretical considerations we hypothesize the following:

Hypothesis 1: Within-person variations in time pressure during daily work should relate curvilinearly to within-person variations in (a) the likelihood to activate thought control to stop distracting thoughts and (b) the effort that is mobilized to stop distracting thoughts in an inverted u-shaped way.

Hypothesis 2: Within-person variations in task complexity during daily work should relate curvilinearly to within-person variations in (a) the likelihood to activate thought control to stop distracting thoughts and (b) the effort that is mobilized to stop distracting thoughts in an inverted u-shaped way.

## Ability to Suppress Unwanted Thoughts

As the points at which challenge demands motivate or hinder individuals’ thought control seems to depend on dispositional cognitive resources, we also investigated interindividual differences in the ability to suppress unwanted thoughts (Levy and Anderson, 2008). The executive deficit hypothesis (Levy and Anderson, 2008) proposes that differences in the ability to suppress unwanted thoughts stem, in part, from differences in executive control abilities such as inhibition that has been supported by neurocognitive (e.g., Anderson, 2004; Anderson and Huddleston, 2012) and behavioral studies (e.g., Levy and Anderson, 2008). Executive control processes such as inhibition are necessary for purposeful, intelligent thinking and acting (Kane and Engle, 2002; Levy and Anderson, 2008), and are related to working memory capacity (Delaney and Sahakyan, 2007; Aslan et al., 2010; Fawcett and Taylor, 2012).

We assume that workers with a higher ability to suppress distractive thoughts might have more cognitive capacity to activate thought control in a given situation and, consequently, do not have to put much effort in the attempt to exert thought control. Thus, we propose the following:

Hypothesis 3: The ability to suppress unwanted thoughts should relate positively to (a) the likelihood to activate thought control to stop distracting thoughts and (b) negatively to the effort that is mobilized to stop distracting thoughts.

We assumed that individuals who are able to suppress distractive thoughts will be less vulnerable to high challenge demands as compared to individuals with lower abilities. Thus, they have enough executive control resources left to activate thought control even under high time pressure or while performing a highly complex task. Therefore, we predict the following:

Hypothesis 4: The ability to suppress unwanted thoughts should moderate the curvilinear relationship between time pressure and (a) the likelihood to activate thought control to stop distracting thoughts and (b) the effort that is mobilized to stop distracting thoughts in such a way that the inflection point after which the relationship turns negative occurs on higher levels of time pressure when the ability to forget is high compared to a low ability to forget.

Hypothesis 5: The ability to suppress unwanted thoughts should moderate the curvilinear relationship between task complexity and (a) the likelihood to activate thought control to stop distracting thoughts and (b) the effort that is mobilized to stop distracting thoughts in such a way that the inflection point after which the relationship turns negative occurs on higher levels of task complexity when the ability to forget is high compared to a low ability to forget.

## Materials and Methods

### Sample

Following the recommendations of Simmons et al. (2011), we have determined a sample size of 150 persons prior to data collection due to practical reasons. The study was time consuming for the participants: Workers were asked to respond to a general questionnaire, to participate in the think-no think task (TNT task) in the laboratory, which lasted 2.5 h, and finally to participate in an experience sampling study (three times on five working days).

To recruit participants, we distributed study information via professional online networks, social media (e.g., xing.com), and flyer in public organizations (e.g., libraries) and in companies. One hundred sixty-seven workers agreed to participate. One hundred sixty-four workers came to our laboratory, and 162 workers finished the think/no-think task successfully. During the experience sampling week, 158 workers completed at least two questionnaires, of whom 143 reported distractive thoughts at least once, and 124 twice or more. As it was difficult to predict how many questionnaires a participant would respond to, our last recruitment initiative yielded seven participants less than sought. Participants received EUR 50 for their participation.

In the final sample of 158 workers (57% female), individuals were between 18 and 71 years old (*M* = 36.56 years, *SD* = 12.27 years). They worked in different industries, primarily performed office jobs, and worked 40.57 h/week on average (*SD* = 7.79 h). They were all able to speak German at a native speaker level and had normal or corrected-to-normal vision. During the experience sampling, the participants completed a total of 1,565 questionnaires (*M* = 9.91, *SD* = 3.16; range, 2–15). In the following analyses, sample sizes vary according to the outcome variable (*n* = 158 for distractive thoughts, *n* = 143 for the likelihood to activate thought control to stop distractive thoughts, and *n* = 91 for the effort to stop distractive thoughts).

### Procedure

Our study consisted of three parts. First, workers filled out a general online questionnaire 1 week prior to their laboratory appointment. Second, after signing a consent form, workers were asked to perform the TNT task in the lab (for more details, see below). After the end of the TNT task, workers were informed about the content, procedure, and technical aspects of the experience sampling study. They were instructed to respond to the three questionnaires per day on five consecutive working days in a typical working week on a smartphone app immediately after noticing the signals sent by the app. To schedule the measurement points, participants were asked to provide information on their typical work week, working hours, and lunch breaks. Lastly, they filled out a practice questionnaire on their smartphone. During the experience sampling, the application sent short beeps or vibrations three times a day. The signals were sent randomly during participants’ individual working hours, but not during their lunch break, and with a minimum time lag of 80 min. Workers had up to 10 min to start their response. After completing the experience sampling, workers were debriefed.

### Measures

In the general questionnaire, we assessed demographic variables (gender, age, educational level, working hours, industry) and psychological health (depression, anxiety). In the laboratory, the ability to suppress unwanted thoughts was assessed with the TNT task. In the experience sampling part, we measured momentary time pressure, momentary task complexity, the outcome variables activation of thought control, and effort to stop distractive thoughts.

#### Ability to Suppress Unwanted Thoughts

The ability to suppress unwanted thoughts has been extensively studied in the laboratory with the think/no-think task (Anderson and Green, 2001). In this task, individuals are trained to suppress target words (“no-think”) that are associated with words they had previously learned. A typical result pattern is that individuals recall fewer suppressed words than controls for which no suppression instructions had been received and even fewer suppressed words than words they are instructed to remember (“think”).

In the current study, we used 58 weakly related, neutrally valenced word pairs from a standardized German database (Melinger and Weber, 2016). Ten word pairs were included as fillers and for training purposes; the remaining 48 were divided into three groups and rotated across experimental conditions (baseline, think, no-think) and across subjects (counterbalancing groups A–C). All word pairs comprised exclusively nouns, with the left noun always the cue and the right noun always the response word. They were presented in the center of a computer screen on a white background using the E-Prime 2.0 software (Psychology Software Tools Inc., Sharpsburg, MD, United States) and separated from the subsequent pair by the short insertion of a fixation cross. The TNT task is structured into four phases (Anderson and Green, 2001). In the first phase (*learning*), workers studied all 58 word pairs. Each word pair was presented in black for 6,000 ms in random order. In the second phase (*recall*), participants were asked to freely recall the response words and say them out loud. For this, each cue was presented for up to 4,000 ms in a pseudo-randomized order. The correct answer was then presented for 1 s immediately following the participant’s response, giving participants the opportunity to check whether their answer was correct. If a worker remembered <50% of the word pairs, we repeated the recall phase. After that, we assessed which of the word pairs had been learned with the same procedure (presenting time up to 3,300 ms), but did not give feedback in order to prevent further unrecorded learning.

The third phase (*think/no-think*) was the main part of the task. The cues were presented for 3,000 ms and the participants’ task varied depending on word color: Think cues appeared in green. Here, participants were to further rehearse the related response word in silence in order to foster the connection. No-think cues appeared in red. Here, participants were asked to immediately stop all thoughts about the response word and not to think about the connections they had made during the learning and recall phases. They were further instructed to only focus on the presented no-think cue and to block out any related thoughts without distracting themselves (*direct suppression* instructions). During the subsequent actual think/no-think phase, each of the 16 think and 16 no-think cues was presented 12 times. The presentation order was determined randomly. To counteract computer eyestrain and tiredness, we incorporated three short breaks (45 s).

In the last phase (*recall*), workers were shown all cue words (baseline, think, no-think) again in a pseudo-randomized order. The words were presented in black for up to 3,300 ms, and participants were asked to recall the associated response word out loud irrespective of the instructions in the think/no-think phase. The difference in recall rates between the baseline condition and the no-think condition was used as an indicator of individuals’ ability to suppress unwanted thoughts. The higher a person’s score on this indicator, the higher their ability to suppress memory retrieval.

#### Distractive Thoughts

A questionnaire in the experience sampling began by asking whether the workers had had any disruptive or distracting thoughts during the past 30 min (*distractive thoughts*, “Did you have to think of something that disrupted and distracted you at work in the last 30 min?;” “no” coded as 0 and “yes” coded as 1).

#### Activation of Thought Control

If the participants experienced an distractive thought, they were also asked whether they had activated thought control to stop distracting thoughts (*activation of thought control*, “Did you say something to yourself like ‘I do not want to think about this anymore’ or ‘I want to forget that’?;” “no” coded as 0 and “yes” coded as 1).

#### Effort to Stop Distractive Thoughts

If workers reported such an attempt, we further asked about their *effort to stop distractive thoughts* (“How much did you try to suppress the distracting thoughts?”). Participants answered on a 5-point rating scale ranging from 1 (*not at all*) to 5 (*extremely*).

#### Time Pressure

After the questions about distractive thoughts, and subsequent actions, we asked about *time pressure* with a single item (“I have had little time to do my job in the last 30 min;” adapted from Spector and Jex, 1998). Responses ranged on a 5-point scale (1 = *not at all* to 5 = *extremely*).

#### Task Complexity

In addition, we assessed *task complexity* with a single item (“The task I have been working on for the past 30 min is complex;” adapted from Maynard and Hakel, 1997). Participants answered on a 5-point rating scale ranging from 1 (*not at all*) to 5 (*extremely*).

As control variables, we assessed strength of distraction by asking “How much did you feel distracted by these thoughts?” (5-point scale 1 = *not at all* to 5 = *extremely*). In addition, we measured depression with 20 items of the Beck Depression Inventory (Schmitt et al., 2006; Cronbach’s alpha = 0.90), and anxiety with the five-item short-scale STAI-SKD (Englert et al., 2011; Cronbach’s alpha = 0.82). Studies have shown that depressed or anxious persons had a lower ability to suppress unwanted thoughts, which can be explained by downsized brain activity in the dorsolateral prefrontal cortex (Anderson et al., 2004; Benoit and Anderson, 2012). This area is usually needed and activated during inhibition processes. In addition, we assumed that these persons should be less motivated to engage in thought control when they experienced distraction.

## Results

### Preliminary Analyses

Before testing the hypotheses, preliminary analyses with respect to the measurement of the ability to suppress unwanted thoughts, and the measures of the experience sampling part of the study were conducted.

#### Ability to Suppress Unwanted Thoughts

To assess a score for the ability to suppress unwanted thoughts, first, the performance data for the TNT task had to be analyzed. We used a mixed-design analysis of variance (ANOVA) with response condition (baseline, think, no-think) as the within-person factor and counterbalancing condition (A–C) as the between-person factor. As recommended by Anderson (2004); see also Levy and Anderson, 2012; Streb et al., 2016), we *z*-normalized all values within the participants’ counterbalancing group to make sure our results were not based on differences in memorability between different sets of word pairs. The final test data for the TNT task revealed that recall rates in the three conditions (no-think, think, baseline) differed significantly from one another [*F*(2,155) = 60.58, *p* < 0.001, partial η^2^ = 0.28]. Contrast comparisons showed a significant suppression-induced forgetting effect: no-think items (*M* = 0.84, *SD* = 0.19) were more poorly recalled than baseline items [*M* = 0.94, *SD* = 0.08; *F*(1) = 45.51, *p* < 0.001, η = 0.23], indicating that participants successfully suppressed memory retrieval. In addition, the recall rates of the no-think items and baseline items were lower than the recall rates of the think items [*M* = 0.97, *SD* = 0.07*;* no-think*: F*(1) = 90.51, *p* < 0.001, η = 0.37; baseline: *F*(1) = 18.40, *p* < 0.001, η = 0.11]. There were no differences between the three item counterbalancing groups, *F*(2,155) = 0.05, *p* = 0.955, partial η^2^ = 0.00, which indicates that the lower recall rates for the no-think items are not influenced by specific word combinations.

Then, we computed the difference in recall rates between the baseline condition and the no-think condition as an indicator of individuals’ ability to suppress unwanted thoughts.

#### Experience Sampling

One hundred fifty-eight participants responded to an average of 9.91 out of 15 surveys (range = 2–15). In total, they completed 1,565 out of 2,370 surveys, leading to a response rate to the signals of 66%. Distractive thoughts were reported in 35.97% of all surveys and thought control activities to stop these thoughts in 14.19% of all surveys (39.43% of distractive thought episodes). Participants reported having distractive thoughts enter their mind in 563 surveys (143 participants) and reported thought control activities to stop the distractive thoughts in 222 surveys (91 participants). Table 1 shows means, standard deviations, and intercorrelations between the study variables at level 2 (person level), while Table 2 provides the same information at level 1 (experience sampling level).

**TABLE 1:** Means, standard deviations, and correlations for level 2 (person-level) variables.

Variable	*M*	*SD*	1	2	3	4	5	6	7	8	9	10
**Person-level variables**												
1. Suppression ability^1^	0.00	0.99										
2. Age^1^	36.56	12.27	−0.19*									
3. Education level^1^	5.35	1.73	–0.01	−0.21**								
4. Depression^1^	2.18	0.70	–0.02	0.04	–0.05							
5. Anxiety^1^	1.91	0.56	0.10	0.04	0.03	0.63**						
**Experience sampling variables**												
**(aggregated on the person level)**												
6. Time pressure^1^	2.27	0.68	0.12	–0.07	0.08	0.00	0.01					
7. Task complexity^1^	3.01	0.65	0.01	0.09	–0.02	−0.17*	−0.20*	0.34**				
8. Distractive thoughts^1^	0.37	0.23	0.08	−0.18*	–0.02	0.37**	0.26**	0.29**	0.07			
9. Strength of distractive thoughts^2^	2.77	0.73	0.00	–0.01	0.05	0.30**	0.33**	0.11	0.03	–		
10. Stopping distractive thoughts^2^	0.36	0.35	0.08	–0.10	0.05	0.06	0.09	0.17*	0.19*	–	0.21*	
11. Effort to stop distractive thoughts^3^	3.12	0.68	0.01	0.16	–0.11	0.01	–0.05	–0.05	0.35**	–	0.07	–

^1^*n* = 158. ^2^*n* = 143. ^3^*n* = 91. *p < 0.05; **p < 0.01 (two-tailed).

**TABLE 2:** Means, standard deviations, and correlations for level 1 (experience sampling) variables.

Variable	*M*	*SD*	1	2	3	4	5
**Experience sampling variables**							
1. Time pressure^1^	2.26	1.09					
2. Task complexity^1^	3.00	1.04	0.13**				
3. Distractive thoughts^1^	0.36	0.48	0.20**	–0.01			
4. Strength of distractive thoughts^2^	2.90	1.01	0.21**	0.03	–		
5. Stopping distractive thoughts^2^	0.39	0.49	0.04	0.10*	–	0.16**	
6. Effort to stop distractive thoughts^3^	3.15	0.80	0.01	0.17*	–	0.16*	–

^1^*n* = 1,565. ^2^*n* = 563. ^3^*n* = 222. *p < 0.05; **p < 0.01 (two-tailed).

We used the R software^1^ and the *lme4* package (Bates et al., 2015) to conduct multilevel analyses. Level 1 variables from the experience sampling study were centered around the person mean in order to consider situational fluctuations, whereas level 2 variables were grand-mean centered. Between-level variance was analyzed by calculating intraclass coefficients (ICC_1_) for all outcome variables. We accounted for the nesting of observations in persons and also allowed for random slopes in all level 1 variables [strength of distractive thoughts, time pressure (linear and squared), task complexity (linear and squared)]. Following the recommendations of Bates et al. (2018), we specified the random effects structure with the diagonal matrix.

The ICC_1_ values were 0.24 for activation of thought control, 0.24 for effort to stop distractive thoughts, and 0.13 for distractive thoughts. Thus, there was sufficient level 1 variance in the outcome variables to justify multilevel modeling. Furthermore, we checked the mode assumptions for the generalized linear and linear mixed effects models using graphic residual plots as recommended in the literature (Bolker et al., 2009). Results revealed no problematic deviations from normality in the residuals.

### Hypotheses Testing

Logistic multilevel modeling conducted with data from 158 participants [*n*(level 1) = 1,565) revealed that the frequency of reporting distractive thoughts [γ = 0.42, odds ratio (OR) = 1.53, *z* = 4.46, *p* < 0.001] increased with increasing time pressure. Table 3 shows the results of the final logistic multilevel model testing the relationship between challenge demands (time pressure, task complexity) and the outcome variable activation of thought control. For reasons of clarity, we included both time pressure and task complexity in our models. Calculating separately lead to the same results. The model includes only occasions in which individuals reported experiencing a distractive thought [*n*(level 1) = 563, *n*(level2) = 143]. In all subsequent multilevel analyses, we held the strength of distractive thoughts constant by including it as control variable. Additionally controlling for age, educational level, depression, and anxiety did not change the results. Contrary to Hypotheses 1a,b and 2a, b, the results showed no significant curvilinear relationships between either challenge demand and the dependent variable thought control activities to stop the distractive thoughts (time pressure: γ = −0.19, OR = 0.83, *z* = −1.80, *p* = 0.071; task complexity: γ = 0.03, OR = 1.03, *z* = 0.21, *p* = 0.837). However, we found that thought control activities were more likely with linearly increasing task complexity (γ = 0.32, OR = 1.38, *z* = 2.11, *p* = 0.035). In addition, the strength of the distractive thoughts predicted the likelihood of a person trying to stop them (thought control activities, γ = 0.44, OR = 1.55, *z* = 3.06, *p* = 0.002).

**TABLE 3:** Predictors of the intention to stop distractive thoughts and the effort needed to stop them.

	Stopping distractive thoughts^1^				Effort needed to stop distractive thoughts^2^			
Predictor	*b*	95% CI	*z*	*b*_cs_	*b*	95% CI	*t*	*b*_cs_
Constant	–0.51	[−0.89, −0.13]	−2.64**	–0.51	3.08	[2.91, 3.24]	37.32**	–
Strength of distractive thoughts	0.44	[0.16, 0.72]	3.06**	0.34	0.10	[−0.06, 0.27]	1.28	0.10
Suppression ability	0.53	[0.10, 0.96]	2.40*	0.53	0.10	[−0.08, 0.29]	1.14	0.13
Time pressure	–0.08	[−0.35; 0.19]	–0.57	–0.07	0.02	[−0.11, 0.15]	0.32	0.02
Task complexity	0.32	[0.02, 0.62]	2.11*	0.27	0.03	[−0.11, 0.17]	0.45	0.03
Time pressure ^∧^ 2	–0.19	[−0.39, 0.02]	−1.80†	–0.14	0.02	[−0.10, 0.13]	0.29	0.01
Task complexity ^∧^ 2	0.03	[−0.25, 0.31]	0.21	0.02	0.06	[−0.05, 0.17]	1.02	0.05
Time pressure × suppression ability	0.27	[−0.05, 0.60]	1.64	0.24	0.04	[−0.12, 0.19]	0.47	0.04
Task complexity × suppression ability	0.03	[−0.26, 0.32]	0.20	0.02	–0.02	[−0.17, 0.13]	–0.24	–0.02
(Time pressure ^∧^ 2) × suppression ability	–0.32	[−0.62, −0.02]	−2.08*	–0.24	–0.16	[−0.30, −0.01]	−2.13*	–0.15
(Task complexity ^∧^ 2) × suppression ability	–0.17	[−0.46, 0.12]	–1.15	–0.12	–0.04	[−0.18, 0.09]	–0.64	–0.04

Continuous level 1 variables (strength of distractive thoughts, time pressure, task complexity) were centered around the person mean; suppression ability (level 2) was *z*-normalized at the grand mean. Coefficients with standardized continuous variables (*b*_*cs*_) were derived by additionally centering all continuous level 1 variables at the sample mean and setting the standard deviation to 1 (cf. Hox et al., 2018). Random effects (variances), stopping distractive thoughts: strength of distractive thoughts, 0.000; time pressure, 0.000; task complexity, 0.000; time pressure ^∧^ 2, 0.000; task complexity ^∧^ 2, 0.169. Random effects (variances), effort needed to stop distractive thoughts: Strength of distractive thoughts: 0.108; time pressure, 0.000; task complexity, 0.026; time pressure ^∧^ 2, 0.002; task complexity ^∧^ 2, 0.000. ^1^*n*_*Level*__2_ = 143, *n*_*Level*__1_ = 563. ^2^*n*_*Level*__2_ = 91, *n*_*Level*__1_ = 222. ^†^*p* < 0.10, *p < 0.05, **p < 0.01 (two-tailed). CI, confidence interval.

We also found that the ability to suppress unwanted thoughts (dispositional factor) made it more likely that workers activated thought control to stop the distractive thoughts, supporting Hypothesis 3a. There was no relationship between the ability (dispositional factor) and the dependent variable effort to stop distractive thoughts (Hypothesis 3b).

**FIGURE 2:**
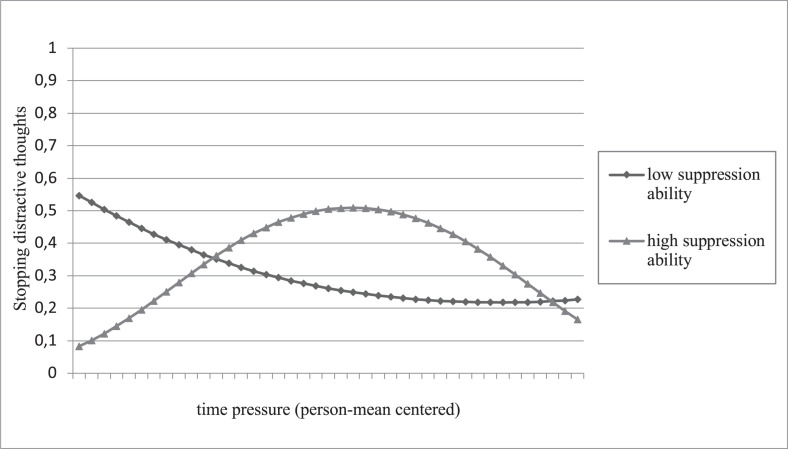
Interaction effect of time pressure and the ability to suppress unwanted thoughts on stopping distractive thoughts (activation of thought control). Higher and lower levels of suppression ability represent one standard deviation above and below the mean.

Next, the data revealed that the ability to suppress unwanted thoughts moderated the curvilinear relationship between time pressure and activation of thought control (γ = −0.32, OR = 0.73, *z* = −2.08, *p* = 0.038), as expected in Hypothesis 4a, but not the relationship between task complexity and activation of thought control (γ = −0.17, OR = 0.85, *z* = −1.15, *p* = 0.251), not supporting Hypothesis 5a. We generated a plot of the interaction at high levels (1 *SD* above zero) and low levels (1 *SD* below the zero) of the ability to suppress unwanted thoughts (see Figure 2). Figure 2 shows that high-ability persons became more likely to activate thought control to stop distractive thoughts with increasing time pressure up to a certain point. However, the relationship turned negative when time pressures increased further. Individuals with a lower ability to suppress unwanted thoughts were more likely to activate thought control when time pressure was below average.

**FIGURE 3:**
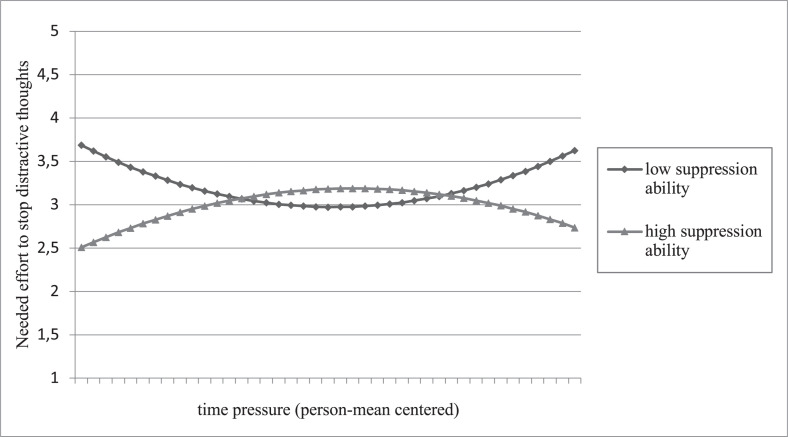
Interaction effect of time pressure and the ability to suppress unwanted thoughts on the effort to stop distractive thoughts. Higher and lower levels of suppression ability represent one standard deviation above and below the mean.

We also proposed a curvilinear relationship between challenge demands and the conscious effort needed to stop distractive thoughts that enter the mind (see Table 3). It is important to note that this model includes only occasions in which individuals reported activation of thought control [*n*(level 1) = 222, *n*(level2) = 91]. We found no significant direct effects (time pressure: γ = 0.02, *t* = 0.29, *p* = 0.774; task complexity: γ = 0.06, *t* = 1.02, *p* = 0.311). The moderation between the ability to suppress unwanted thoughts and time pressure (γ = −0.16, *t* = −2.13, *p* = 0.039), however, reached significance, supporting Hypothesis 4b. Figure 3 shows that individuals with a higher ability to suppress distractive thoughts tried harder to stop unwanted thoughts as time pressure increased but that this relationship turned negative at higher levels of time pressure. In contrast, individuals with a lower ability to suppress distractive thoughts tried harder when time pressure was either low or very high. We found no moderation of the curvilinear relationship between task complexity and effort (γ = −0.04, *t* = −0.64, *p* = 0.525). We found no significant interaction effect between task complexity and the ability to suppress unwanted thoughts predicting the effort to stop distractive thoughts. Thus, Hypothesis 5b was not supported.

## Discussion

Stopping distractive thoughts can be very difficult but is crucial for staying focused. The positive effect of self-control activities on workers’ affective and behavioral responses in the organizational setting is well established as it has been shown in many studies (e.g., Lian et al., 2017). We examined person–situation interactions to understand when workers activate thought control to stop distractive thoughts in daily working life, an underresearched issue in the self-control literature. Thereby, for being precise, we investigated and measured the ability to inhibit unwanted thoughts as one important predictor rather than broader concepts such as self-control, which comprises different aspects (e.g., persistence, impulse control etc.).

As expected, whether time pressure activates or hinders thought control activities depends on individuals’ ability to suppress intrusions. Workers who were skilled at suppressing intrusions stopped distractive thoughts at moderate levels of time pressure more likely, but not at very low or very high time pressure. Likewise, we found some evidence that these workers put less effort into suppressing distractive thoughts when time pressure was low or high. For individuals with a lower ability to suppress distractive thoughts, increasing time pressure was negatively related to thought control activities. Thus, the notion that moderate levels of challenge demands motivate self-control might only be true for individuals who have the relevant abilities as well as, relatedly, sufficient cognitive capacity. Likewise, well-intentioned advice on concentration and time management from the literature, such as “nothing sharpens the attention better than demanding work and the sprint toward a deadline,” do nothing to support individuals who have difficulty stopping irrelevant thoughts when trying to stay focused at work.

Several potential mechanisms may explain the interaction between time pressure and the ability to suppress unwanted thoughts. In line with activation theory (Gardner, 1986; Gardner and Cummings, 1988), workers who were skilled in suppressing unwanted thoughts engaged in less thought control at very high levels of time pressure. One explanation might be that task performance under high time pressure consumes all processing resources that would otherwise be directed to suppression (e.g., Román et al., 2009; Ortega et al., 2012). For example, if a journalist has to finish an article under high time pressure to a certain deadline, he or she has to concentrate completely on the writing process. There is then no capacity left to consciously banish from memory disturbing thoughts such as “I have another article to finish today.” Alternatively, one could suggest that at very high levels of time pressure, workers just switch their strategy in order to cope with the overtaxing memory load: To stay with the example, the journalist devotes then his or her full concentration to the current task in order to keep distracting thoughts at bay. By doing so, he or she did not have to intentionally inhibit unwanted thoughts (for similar findings, see Noreen and de Fockert, 2017). A third mechanism might be that individuals deliberately weigh the benefits of stopping distractive thoughts (e.g., better performance) against its resource costs (mental exhaustion; Kool et al., 2017). Under high time pressure, when it becomes increasingly effortful to stop distractive thoughts, the costs might outweigh the benefits, leading individuals to change deliberately their self control strategy for coping with these high demands.

At very low levels of time pressure, high ability individuals’ activation levels were too low for them to engage in thought control. Alternatively, individuals might have sufficient cognitive capacity to engage in task performance, meaning that distractive thoughts do not distract from the task at hand or are a welcomed interruption to make the task less monotonous or boring. Moreover, as we only studied deliberate thought control that raised sufficient awareness to justify reporting, it might also be possible that these workers dealt with the distractive thoughts so quickly that they were less aware of them.

Also in line with the predictions of activation theory and resource models is that thought control activities already became compromised at lower levels of time pressure for workers who were less able to suppress unwanted thoughts. These workers are only activated to stop distractive thoughts and to put effort into this cognitive activity at very low levels of time pressure.

Despite these interaction effects, the data revealed also direct relationships. As one would expect, the strength of distractive thoughts made thought control activities more likely. In addition, workers with a higher ability to suppress unwanted thoughts engaged more often in thought control. It is also important to note that task complexity was positively related to the thought control activities to stop distractive thoughts, but we found no significant interaction effects with the ability to suppress unwanted thoughts.

In sum, our findings contribute to theory and research on self-control in at least two ways. First, the study showed that moderate levels of challenge demands such as time pressure activate workers’ thought control even if time pressure possibly causes also cognitive costs such as heightened information processing. Thus, it seems that not every competing activity that relies on executive control capacity is compellingly detrimental to the activation of thought control. Second, it depends on individuals’ ability to suppress unwanted thoughts to what extent individuals are activated by challenging demands to engage in thought control to stop distractive thoughts. Therefore, the activation of these self-control activities depend on both situational factors as well as on personal factors. In the present study, workers with a lower ability to control unwanted thoughts had difficulties to engage in thought control activities even at low time pressure. Thus, to stay concentrated to the task at hand, these workers should be supported by trainings to enhance their self-control skills, which can protect against the adverse effects of challenging situational demands. There is already evidence that practicing inhibitory control helps to manage intrusive experiences (e.g., Hulbert and Anderson, 2018; for meta-analytic evidence, see Friese et al., 2017). Thereby, in a typical training session, individuals are asked to repeatedly control dominant responses by, for instance, performing the Stroop task or completing everyday activities with the non-dominant hand (using the computer mouse).

Interventions that aim to structure workers’ tasks by providing intelligent assistance systems for hiding irrelevant digital information on the computer screen might particularly help these workers focus on their current goals when facing time pressure. For example, the assistive system hides folders and files that are associated with competing project that is not in focus at the moment (Siebers et al., 2017). Support might be also provided by time management tools or by allowing employees to accomplish tasks in a quiet and undisturbed work environment.

## Limitations and Implications for Future Research

Despite combining a laboratory task, which measures the ability to inhibit unwanted thoughts, and an experience sampling study, we have to acknowledge several limitations. First, in the experience sampling study, workers reported their thought control activities, which was the focus of the present study. It remains open whether these activities were successful, which would have been an additional interesting information. However, measuring success of stopping distractive thoughts was not feasible with the study’s design. In the experience sampling study, repeatedly asking whether a person has really forgotten a certain memory content might threaten validity and probably provoke an opposite effect after the first question, namely, that the memory contents are even better remembered. In addition, one could argue that the participants faked their responses in the TNT task to be a “good participant.” However, previous research has shown that the effects of retrieval suppression (forgetting) are not due to the intentional withholding of to-be-forgotten items. For example, Macrae and MacLeod (1999) demonstrated that a monetary incentive for the recall of to-be-forgotten items did not increase the recall rates of these items (see also Anderson and Green, 2001). Second, we did not assess the potentially mediating processes between challenge demands, the ability to suppress unwanted thoughts, and the outcome variables. To explain our results, we relied on different mechanisms such as activation, resource allocation, and cost–benefit decisions, which should be further explored in future studies. Third, our concurrent measurement of predictor (challenge demands) and outcome (thought control activity and effort) variables in the experience sampling study limits the causal interpretation of the data. We cannot completely rule out that the experience of distractive thoughts biased the assessment of challenge demands such as time pressure and task complexity. However, correlations among these predictor and outcome variables were rather low. Moreover, our main hypotheses focused on interactive effects and curvilinear effects, which make it difficult to determine how the experience of distractive thoughts should have biased the curvilinear effects. Finally, with respect to common method bias, interactions are generally less affected (Evans, 1985; Siemsen et al., 2010).

## Conclusion

Our findings highlight the importance of examining factors that motivate or hinder workers from engaging in thought control activities to stop distracting thoughts in daily working life. Challenge demands are important to activate workers’ thought control strategies such as stopping distractive thoughts, but can become detrimental quite fast when individuals do not have the appropriate cognitive abilities. The study underlines the importance to examine person–situation interactions to understand how workers stay focused in modern work environments.

## Data Availability Statement

The original contributions presented in the study are included in the article/supplementary files, further inquiries can be directed to the corresponding author.

## Ethics Statement

The studies involving human participants were reviewed and approved by Deutsche Gesellschaft für Psychologie. The patients/participants provided their written informed consent to participate in this study.

## Author Contributions

CN was responsible for study design, supporting data acquisition, and analysis, and writing up the manuscript. KG analyzed the data and supported writing the manuscript. JL supervised and supported the data analysis. US contributed to the study design. All authors contributed to the article and approved the submitted version.

## Conflict of Interest

The authors declare that the research was conducted in the absence of any commercial or financial relationships that could be construed as a potential conflict of interest.

## Footnotes

1 http://r-project.org
